# Review of methods for the determination of cellular cholesterol content: principles, advantages, limitations, applications, and perspectives

**DOI:** 10.1007/s44211-025-00842-5

**Published:** 2025-08-27

**Authors:** Zihang Zhou, Jiangyu Zong, Ning Xu

**Affiliations:** Institute of Drug Development and Chemical Biology, College of Pharmaceutical Science, Zhejiang University of Technology, Huzhou, 313200 Zhejiang People’s Republic of China

**Keywords:** Cholesterol, Biological assay, Probe technology, Cholesterol analogues, Mass spectrometry imaging

## Abstract

Imbalances in cellular cholesterol homeostasis are associated with various diseases, and accurate determination of cholesterol levels and distribution is essential for a thorough understanding of cellular physiopathology. In this review, we comprehensively analyzed various techniques for cellular cholesterol determination. They include indirect methods based on SREBP2 activity monitoring, gas chromatography–liquid chromatography coupling, enzyme analysis, improved Abell–Kendall method, cholesterol-specific probes such as filipin III and cholesterol-dependent cytolysin, and cholesterol analogs such as dehydroergosterol, BODIPY-cholesterol, label-free as well as labeling Raman assays and mass spectrometry imaging techniques such as matrix-assisted laser desorption/ionization mass spectrometry imaging, desorption electrospray ionization mass spectrometry imaging, and nanoscale secondary ion mass spectrometry technology. Principles, advantages, and limitations of each technique are discussed in detail, their characteristics in terms of sensitivity, spatial resolution, and temporal resolution are compared in detail. Finally, suggestions for selecting the technique for different experimental objectives are also given. These findings will help researchers choose the most suitable method according to their own needs, provide strong support for cellular cholesterol research, and promote the development of related fields to better elucidate the significance of cellular cholesterol in normal biologic activities and also its intrinsic relationship with diseases.

**Graphical abstract:**

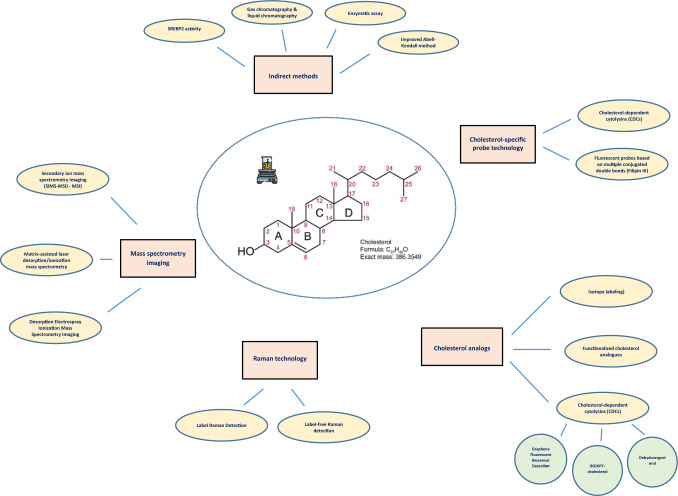

## Introduction

Cholesterol plays an important role in a variety of biological processes as a precursor for the synthesis of steroid hormones, bile acids, and vitamin D [1], and is also a key component of eukaryotic cell membranes [2]. Cell membranes have a typical phospholipid bilayer structure, with hydrophobic tails buried within the membrane and polar heads exposed to the aqueous environment. Cholesterol is inserted into the lipid bilayer and its polar hydroxyl groups are arranged adjacent to the polar head groups of the phospholipids, thus maintaining the integrity of the membrane and regulating its rigidity and permeability. The composition of lipids in membranes, including the amount of cholesterol, directly affects their physical properties, such as permeability and curvature, and plays an important role in regulating the recruitment and activity of membrane proteins [3].

**Fig. 1:**
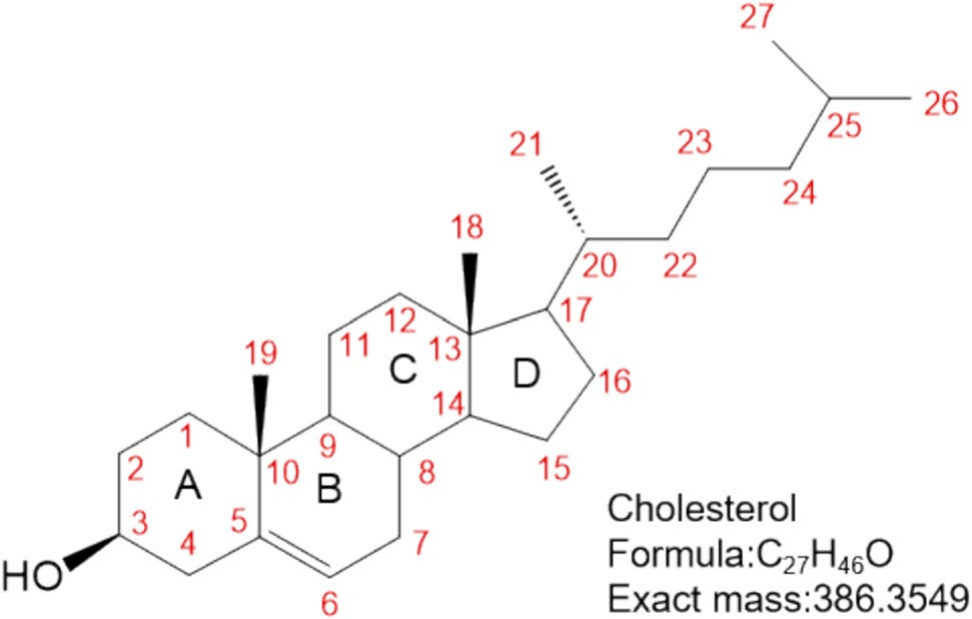
Structure and atomic numbering of cholesterol

The cholesterol molecule is composed of three major components: (1) four carbon rings (A-D) that serve as the steroidal core; (2) a polar hydroxyl group attached to ring A; and (3) a short non-polar carbon chain attached to ring D (Fig. 1) [4,5]. All four sterol rings are in the trans conformation, which gives cholesterol its unique structural stability; the double bond between C5 and C6 helps maintain its rigidity. More than 90% of cellular cholesterol is found in the plasma membrane [6], where its molecules are inserted into the lipid bilayer and arranged in parallel with glycerophospholipids and sphingolipids. The polar hydroxyl group of cholesterol is close to the head of the lipid, while the hydrophobic tail is closely associated with the saturated phospholipids and sphingolipids within the membrane [5]. The plasma membrane contains the highest concentration of cholesterol (30–40 mol% of membrane lipids) [7], which significantly affects membrane rigidity, fluidity, and permeability, and thus plays a key role in maintaining the separation of the cell from the external environment.

Cholesterol exists in two primary forms: free (unesterified) cholesterol and esterified cholesterol, also known as cholesteryl esters. These forms differ structurally, functionally, and in their distribution within the body, with important implications for lipid transport, cellular physiology, and disease processes [5].

Free cholesterol refers to the unesterified form of cholesterol. It is predominantly located on the surface monolayer of lipoprotein particles, including high-density lipoproteins (HDL) and low-density lipoproteins (LDL), as well as within cell membranes where it contributes to membrane fluidity, stability, and the regulation of membrane-bound proteins. Functionally, free cholesterol is essential for maintaining the structural integrity of cell membranes and is involved in processes such as signal transduction and membrane trafficking. Enzymatically, free cholesterol can be esterified by lecithin-cholesterol acyltransferase (LCAT) in the plasma or by acyl-coenzyme A: cholesterol acyltransferase (ACAT) within cells, which converts free cholesterol into cholesteryl esters for transport and storage [5,6].

Cholesteryl esters are formed through the esterification of free cholesterol with a fatty acid, a reaction catalyzed by LCAT or ACAT. This modification renders cholesterol more hydrophobic, allowing cholesteryl esters to be efficiently packed and stored within the hydrophobic core of lipoprotein particles such as LDL and HDL, as well as within intracellular lipid droplets. Cholesteryl esters serve as a key transport and storage form of cholesterol, facilitating its movement through the aqueous environment of plasma and its deposition in tissues. The enzyme cholesteryl ester transfer protein (CETP) mediates the exchange of cholesteryl esters between lipoproteins, a process critical for reverse cholesterol transport and lipoprotein remodeling [1,2].

Free cholesterol and cholesteryl esters exhibit distinct physicochemical properties: free cholesterol remains amphipathic and integrates into lipid monolayers, whereas cholesteryl esters, due to their greater hydrophobicity, localize to the lipid core of lipoproteins. The dynamic equilibrium between these forms is regulated by esterification (via LCAT and ACAT) and hydrolysis reactions, which together maintain cellular and systemic cholesterol homeostasis. Clinically, the accumulation of cholesteryl esters within macrophages and arterial walls contributes to foam cell formation and the development of atherosclerotic plaques, highlighting the significance of their metabolism in cardiovascular disease [1,2,5,6].

Compared to the plasma membrane, the endoplasmic reticulum (ER) membrane contains a lower amount of cholesterol, only 3–6% of membrane lipids. This is because the ER is the primary site of cholesterol synthesis, and newly synthesized cholesterol is rapidly distributed to other organelles or stored in lipid droplets by esterification [2]. This distributional property reflects the tight regulation of intracellular homeostasis by cholesterol. Imbalanced cholesterol levels are often associated with organelle dysfunction and disease [8]. For example, accumulation of cellular cholesterol can not only disrupt the physical properties of membranes, but also indirectly alter the function of signaling pathways by affecting the recruitment and activity of membrane proteins [3,9]. Thus, cholesterol is pivotal in maintaining cell membrane structure, regulating lipid metabolism, and mediating signal transduction.

Therefore, the determination of cholesterol content is of particular importance.

This review systematically examines the current methodologies employed for the detection, quantification, and visualization of cholesterol in biological samples. These approaches can be broadly categorized into indirect measurement methods, cholesterol-specific probe technologies, cholesterol analog-based strategies, Raman spectroscopy techniques, and mass spectrometry imaging modalities.

**Indirect measurement methods** include assays and chromatographic techniques that infer cholesterol levels through enzymatic activity or chemical properties. These encompass cholesterol determination based on sterol regulatory element-binding protein 2 (SREBP2) activity, gas chromatography (GC) and liquid chromatography (LC) approaches, enzymatic colorimetric assays, and improved variations of the classical Abell-Kendall method.

**Cholesterol-specific probe technology** leverages molecules that bind directly or selectively to cholesterol, such as fluorescent polyene probes like Filipin III and cholesterol-dependent cytolysins (CDCs), enabling the visualization of free cholesterol in cells and tissues.

**Cholesterol analogs** serve as powerful tools for tracking cholesterol dynamics, with fluorescent analogs (e.g., dehydroergosterol and BODIPY-cholesterol), isotope-labeled cholesterols, and functionalized derivatives facilitating detailed studies of cholesterol distribution, transport, and metabolism.

**Raman technology** offers both label-free and label-based detection strategies, providing molecular-level information about cholesterol with high spatial resolution, making it suitable for in situ analysis of biological samples.

**Mass spectrometry imaging (MSI)** techniques, including matrix-assisted laser desorption/ionization (MALDI–MSI), desorption electrospray ionization (DESI–MSI), and secondary ion mass spectrometry (SIMS–MSI), enable label-free, high-resolution spatial mapping of cholesterol in tissues, offering unparalleled insights into its localization and accumulation in pathological states.

This comprehensive overview sets the stage for a detailed discussion of each method, highlighting their principles, advantages, limitations, and applications in cholesterol research.

## Indirect measurement methods

### Cholesterol assay based on SREBP2 activity

**Fig. 2:**
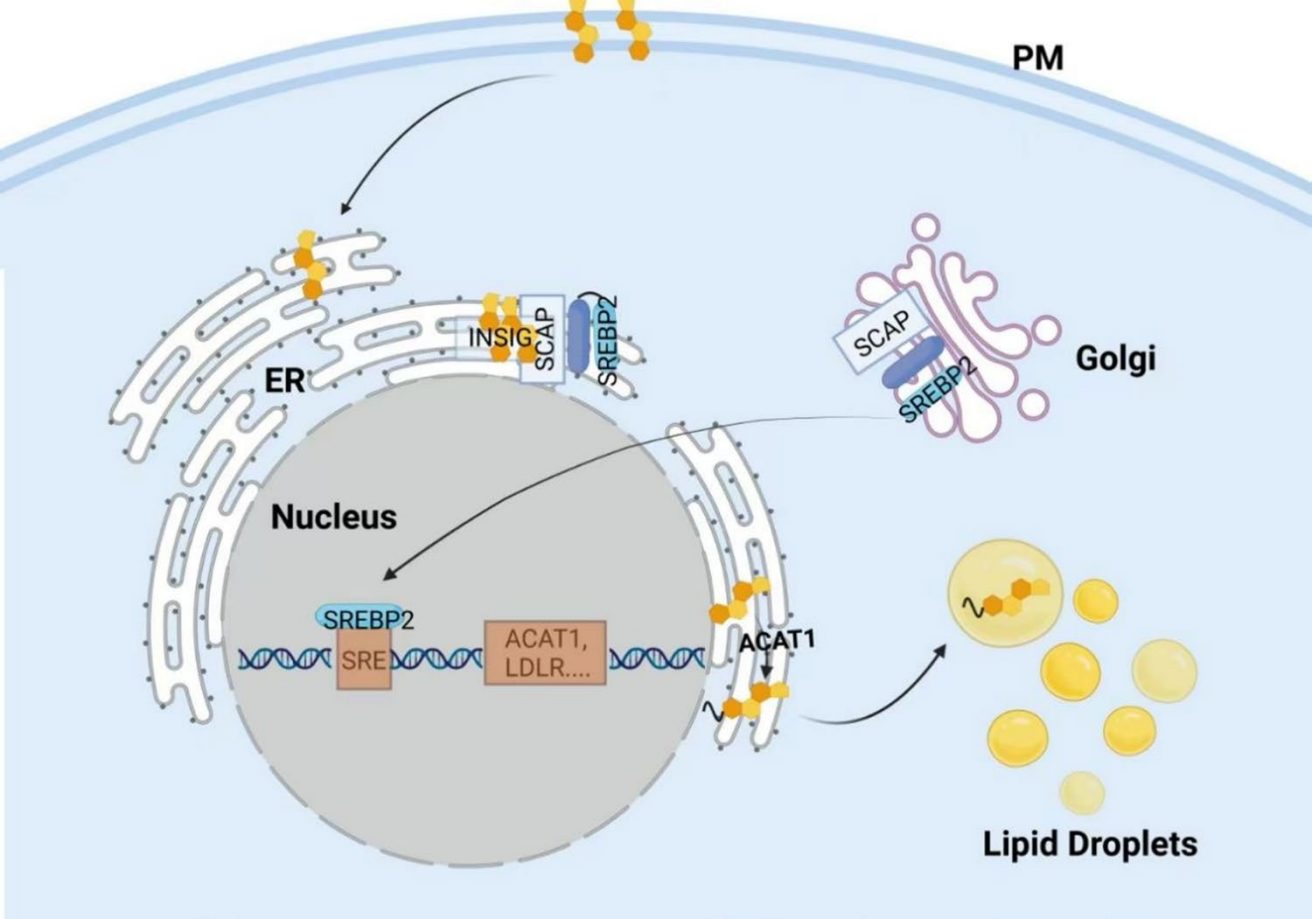
SREBP-dependent steps involved in the cellular response to increasing cholesterol levels in the ER membrane. Sterol sensing by INSIG results in the release of SREBP2 and SREBP cleavage activating protein (SCAP) from the ER membrane, followed by SREBP2 cleavage in the Golgi. In the nucleus, cleaved SREBP2 binds to the sterol recognition element (SRE), leading to the down-regulation of key factors in sterol biosynthesis and sterol uptake, while increasing cholesterol esterification through up-regulation of ACAT1 and subsequent storage in lipid droplets

Sterol regulatory element binding protein 2 (SREBP2) is a key transcription factor in the regulation of cholesterol metabolism and directly controls the transcriptional program of cellular cholesterol biosynthesis [10]. The proposed details are presented in Fig. 2. As a dual transmembrane protein, SREBP2 is synthesized in the ER and then requires activation by Golgi processing. Specifically, proteolytic hydrolysis of SREBP2 releases its N-terminal transcription factor structural domain, which is then translocated to the nucleus where it binds to sterol regulatory elements (SREs) on the promoters of target genes and promotes transcription of genes related to cholesterol metabolism [11]. This process is precisely regulated by SREBP cut activating protein (SCAP) and insulin inducible genes (INSIG). SCAP acts as a chaperone protein for SREBP2 and anchors SREBP2 to the ER by binding to INSIG; upon dissociation of INSIG. The SCAP-SREBP2 complex is translocated to the Golgi and initiates SREBP2 cleavage processing [12,13]. SCAP and INSIG are two sterol-sensing proteins that sense intramembrane cholesterol fluctuations and regulate SREBP2 activity. SREBP2 processing is activated when ER membrane cholesterol levels fall below 5 mol% of total lipids, providing a dynamic cellular response to cholesterol demand [14,15]. The high sensitivity of this process makes SREBP2 an important indicator for monitoring intracellular cholesterol levels.

The full-length form (120–125 kDa) and the processed nuclear form (64–66 kDa) of SREBP2 can be detected by protein blotting (Western blot) due to significant molecular weight differences. Under homeostatic conditions, full-length SREBP2 predominated in cells, whereas the processed nuclear form dominated under sterol depletion conditions. Determining SREBP2 activity provides a simple and effective means of qualitatively assessing the bioavailability of cellular cholesterol [16]. In addition, SREBP2 activity-based reporter systems are a commonly used as a monitoring tool. For example, by clustered regularly interspaced short palindromic repeats (CRISPR)-mediated knock-in of the fluorescent protein trichostatin into the 3-hydroxy-3-methylglutaryl coenzyme A synthase (HMGCS1) motif, an HMGCS1-trichostatin fusion protein was constructed for real-time monitoring of SREBP2 transcription factor activity [17]. Genome-wide CRISPR screening combined with positive or negative feedback regulation of LDLR expression allows systematic screening of important factors involved in cholesterol mobilization [18].

### Cholesterol determination based on gas chromatography, liquid chromatography and mass spectrometry

Gas chromatography (GC) and liquid chromatography (LC) are two chromatographic techniques commonly used for quantitative analysis of cholesterol. These methods are widely used in the determination of cholesterol because of their high sensitivity, accuracy, and reliability. Chromatographic methods provide better selectivity than other analytical techniques, especially since cholesterol can be effectively separated from other interferences (e.g., steroids) in the chromatographic columns, thus improving the specificity of the assay. In addition, the sample volumes required for these methods are usually small (only a few tens of microliters), making them convenient in practice. However, the performance of chromatographic techniques is highly dependent on sample pretreatment, including steps such as saponification and analyte extraction, which can significantly affect the results. GC coupled with a hydrogen flame ionization detector (FID) was first applied for the determination of cholesterol in serum in 1964 [19]. In 1979, reversed-phase high-performance liquid chromatography (HPLC) coupled with ultraviolet detection (wavelength λ = 210 nm) was used for the determination of free cholesterol, esterified cholesterol, and total cholesterol in serum, and a linear range of 0.09–0.31 g/L was obtained [20].

Intracellularly, cholesterol is formed and stored in lipid droplets after esterification catalyzed by the ER-resident acyl-coenzyme A cholesterol acyltransferase (ACAT) [21]. Thus, cholesterol esterification can also serve as a marker for cholesterol transport to the ER. Pulse-tracking assays can be used to monitor the esterification reaction by determining the binding of radiolabeled cholesterol or oleic acid to cholesteryl esters in combination with thin-layer chromatography (TLC) to separate cell-extracted free cholesterol and cholesteryl esters. GC and LC combined with mass spectrometry have been widely used for quantitative analysis of cholesterol and cholesteryl esters. These methods can effectively separate cholesterol from other interferences and have higher sensitivity and reliability compared to enzymatic techniques [22]. However, mass spectrometry analysis often requires sample derivatization to improve the ionization efficiency of cholesterol and isotope-labeled cholesterol internal standards.

In recent years, air-pressure mass spectrometry techniques, such as real-time analytical mass spectrometry (DART), have opened up new possibilities for rapid, low-labor-intensive quantitative analysis of cholesterol, which is useful for rapid screening [23]. Although these mass spectrometry techniques provide effective quantitative analysis tools, they often fail to give information about the spatial distribution of cells or sub-cells. In contrast, matrix-assisted laser desorption/ionization mass spectrometry imaging (MALDI-MSI) and desorption electrospray ionization mass spectrometry imaging (DESI-MSI) techniques can visualize the spatial distribution of cholesterol sulfate or cholesterol directly on tissue sections with a resolution of 30 μm and 200 μm, respectively, which has enabled cholesterol distribution at the tissue level [24].

### Enzymatic assay of serum cholesterol

The first enzymatic assay for the measurement of serum cholesterol was developed in 1974 [25]. Since then, enzymatic assays have gradually become more widely used, with numerous applications in today’s test kits as well as in automated analyzers [26]. In this technique, first, cholesterol esterase hydrolyzes esterified cholesterol to free cholesterol [27]. Next, cholesterol oxidase further oxidizes free cholesterol to cholest-4-en-3-one. This reaction generates hydrogen peroxide as a by-product, which can be easily detected using highly sensitive colorimetric or fluorescent probes. Since many fluorescent probes have been developed, enzymatic assays have become a common detection strategy. The 4-amino antipyrine (known as Trinder’s reagent when phenol is added) [25], homovanillic acid [28], and 10-acetyl-3,7-dihydroxyphenoxazine (also known as Amplex Red) [29] are all known to form fluorescent products catalyzed by the enzyme horseradish peroxidase (HRP) in the presence of hydrogen peroxide. For routine analysis, several laboratories use commercially available quantitative cholesterol test kits based on enzymatic assays, portable point-of-care (POCT) devices, and automated analyzers [26,30]. It should be noted, however, that enzymatic assays may not be completely selective in determining cholesterol levels. Cholesterol oxidase may react with other sterols [31]. In addition, some chemicals present in antioxidants (e.g., ascorbic acid and bilirubin) may consume hydrogen peroxide, which could potentially lead to cholesterol quantification bias when performed indirectly.

### Improved Abell–Kendall method

The Abell–Kendall method [32] was originally proposed in the middle of the twentieth century as a classical chemical-analytical method for the accurate determination of total cholesterol in serum. The procedure of this original method consists of the following steps: first, cholesterol esters are saponified with alcoholic potassium hydroxide to convert them to free cholesterol; then, cholesterol is extracted from the sample with petroleum ether; and finally, the cholesterol concentration is quantified by UV spectrophotometry (detection wavelength of 410 nm) using the Liebermann–Burchard (L-B) reaction for color development. In the L-B reaction, hydroxyl groups in the cholesterol molecule participate in the reaction, resulting in a green coloration product, the intensity of which is proportional to the cholesterol concentration. The unimproved Abell–Kendall method is cumbersome and requires a high level of environmental and laboratory effort, which is not feasible for large-scale application.

**Fig. 3:**
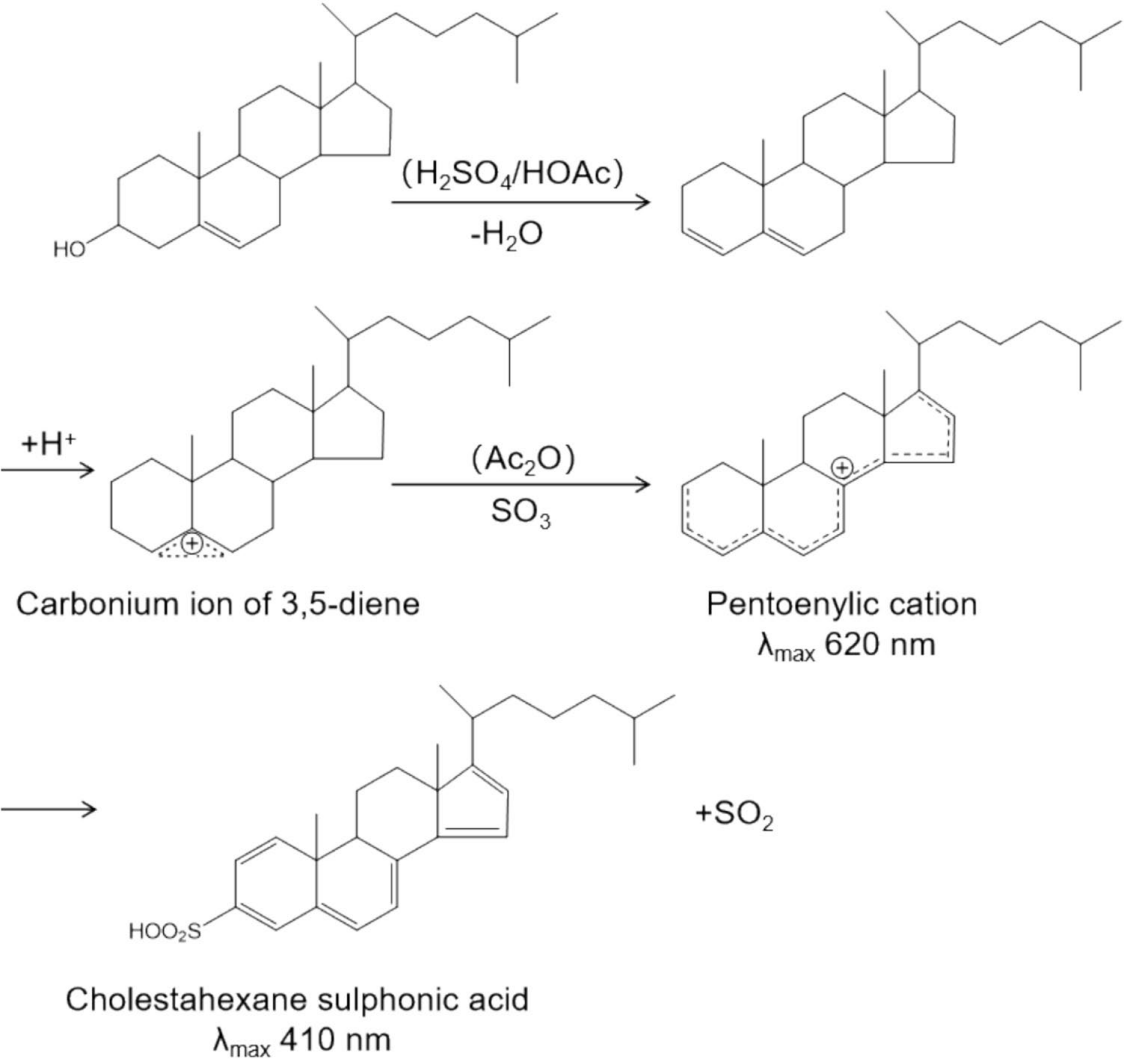
Scheme of the Liebermann–Burchard reaction for color development in the modified Abell–Kendall method

To overcome these problems, researchers modified the technique. Based on the original method, the modified Abell–Kendall method [33] used hexane instead of petroleum ether as the extraction solvent, which greatly improved the safety of the operation; other processes, such as saponification and color development, remained unchanged. This modification improves the practicability of the method while maintaining its high accuracy and reliability as a reference method. The procedure involves saponification of cholesteryl esters with alcohol potassium hydroxide, hydrolysis of cholesterol by hexane extraction, evaporation of solvent, and color development by L-B reaction. The principle of color development is shown in Fig. 3, and the core mechanism is still the chemical reaction of the hydroxyl groups in the cholesterol molecule. Although the modified Abell–Kendall method overcame some of the shortcomings of the original one, it still requires the use of highly corrosive reagents (e.g., concentrated sulfuric acid) in the procedure; therefore, modified Abell–Kendall method has been gradually replaced by safer, simpler, and more modern assays, and is mainly used as a standardized reference method for laboratory research and assay calibration [34].

## Cholesterol-specific probe technology

### Fluorescent probes based on multiple conjugated double bonds (filipin III)

Visualization of the intracellular localization of endogenous cholesterol has been achieved primarily by the use of sterol-binding biomolecules, of which filipin III is the best-known probe. Filipin III specifically interacts with free cholesterol, but does not bind to esterified cholesterol [35]. This specific binding is due to fact that the structure of the filipin molecule contains multiple conjugated double bonds that allows it to form a complex with the hydrophobic portion of the cholesterol molecule. When filipin III binds to free cholesterol, its excitation spectrum peaks at approximately 360 nm, and its emission spectrum peaks at approximately 480 nm. By detecting fluorescence signals near these specific wavelengths, the cholesterol content of the cell can be characterized individually. It is therefore well suited for visualizing free cholesterol without staining the esterified cholesterol stored in lipid droplets [36]. This property has made it an important tool in the study of Niemann–Pick type C disease (NPC) and has greatly advanced the understanding of the cellular mechanisms of the disease [37]. In addition, Wang Hongyan et al. [38] used filipin III to label cholesterol on cell membranes when investigating cholesterol-related mechanisms. They observed the distribution of cholesterol in cells and the changes in its content under different treatment conditions. This clearly showed the dynamic changes in the localization of cholesterol on cell membranes under the action of specific stimuli or intervening factors. Tang et al. [39] cultured THP-1 macrophages to form a foam cell model, and then used filipin III staining to show the changes in intracellular cholesterol and its co-localization with apoA-1. Moreover, Chen et al. [40] co-cultured SH-SY5Y cells and C6 cells in vitro, and used filipin III staining to evaluate the cholesterol levels and cholesterol localization in the cells. By using filipin III staining to evaluate the cholesterol level and cholesterol localization in the cells, the staining method was used to support the investigation of the effects of 27-hydroxycholesterol (27-OHC) on cholesterol homeostasis in neurons. This has provided an important basis for further clarification of the mechanism of 27-OHC in cholesterol metabolism and cholesterol localization.

Although filipin III is a classic cholesterol probe, the exact mechanism of its binding to cholesterol is not fully understood. In addition, it has been shown that filipin III also binds to other lipids, in particular ganglioside GM1 [41]. Special care must be taken when interpreting filipin III staining images in tissues with high levels of GM1 (e.g. neural tissue). In addition, filipin III binding disrupts the bilayer structure of the membrane and may lead to cell lysis, which limits the stability and accuracy of its application in fixed cells. Finally, its low photostability further limits the sensitivity of intracellular cholesterol imaging, making sample handling extremely delicate. Despite the optimal excitation wavelength for filipin III is 360 nm in the UV, it can also be excited with a 405 nm laser, although with reduced signal intensity [42,43].

### Cholesterol-dependent cytolysins (CDCs)

Another class of sterol probes is based on cholesterol-dependent cytolysins (CDCs). Several pathogenic bacteria and fungi have evolved to secrete pore-forming toxins as a means of lysing host cells for their nutrients and defense against phagocytosis. Many of these toxins bind to cholesterol-rich regions of the plasma membrane [44].

Perfringolysin O (Theta toxin) is a highly regarded member of the CDCs family, of which Perfringolysin O (PFO) is the most representative. The application of natural PFO is mostly limited to in vitro studies or labeling of fixed cells due to its cytolytic activity [45–47]. To overcome this limitation, researchers have actively explored various strategies to reduce its toxicity. Ohno-Iwashita et al. succeeded in obtaining MCtheta by partial protein hydrolysis and methylation, and this derivative can be progressively biotinylated to BCtheta, which is effectively used for labeling intracellular cholesterol-accumulating regions [45,48]. Hotze et al. constructed mutant PFO* (Y181A, C459A) can label living cells at low temperatures while avoiding cell lysis [46]; in addition, the research results on the identification of the smallest PFO structural domains should not be ignored, such as PFO-D4, whose recombinant fluorescent markers play an important role in a variety of assays, including super-resolution microscopy to observe the microstructural domains of the plasma membrane as well as microscopy-based compound screening [49]. Subsequent improved mutants with lower binding thresholds of D4H have demonstrated important applications in the study of cellular cholesterol distribution [50], although the effect of intracellular expression on cholesterol homeostasis remains to be explored in depth.

Anthrolysin O (ALO) is also a cholesterol-dependent cytolysin, and its C-terminal domain 4 (ALO-D4) has been widely used in cholesterol studies to measure cell surface cholesterol levels, e.g. to reveal a significant difference in cholesterol levels of human erythrocyte interstitial plasma membranes [51], and the role of related proteins in maintaining PM cholesterol levels in primary mouse hepatocytes [52]. Cell surface cholesterol distribution can also be effectively observed using specific methods such as heavy isotope labeling with 15N-ALO-D4 and Nano SIMS imaging [53]. Notably, ALO-D4-bound cholesterol was trapped at the plasma membrane, a property that provides a novel research idea and tool for in-depth study of intracellular transport processes [54]. Ostreolysin A (Oly A), extracted from the flat mushroom, binds cholesterol- and sphingomyelin-containing liposomes, and after recombinant expression and purification, it can be used in live cell studies to detect specific cholesterol regions [55], and binds as well as stabilizes specific microstructural domains that can be used as a tool to trap PM cholesterol [56]. Theonellamides is a double cyclic peptide derived from Marine sponge [16], which can bind to cholesterol as low as 10 molar%. Its fluorescent derivative can recognize cellular cholesterol, which has the potential for observing cholesterol distribution; however, the difficulty of isolation limits its application [57]. The continuous development of CDCs in cholesterol research has provided a key tool for an in-depth understanding of cholesterol-associated physiopathological processes and has advanced in related field of research.

## Cholesterol analogs

### Fluorescent analogs

#### Dehydroergosterol (DHE)

**Fig. 4:**
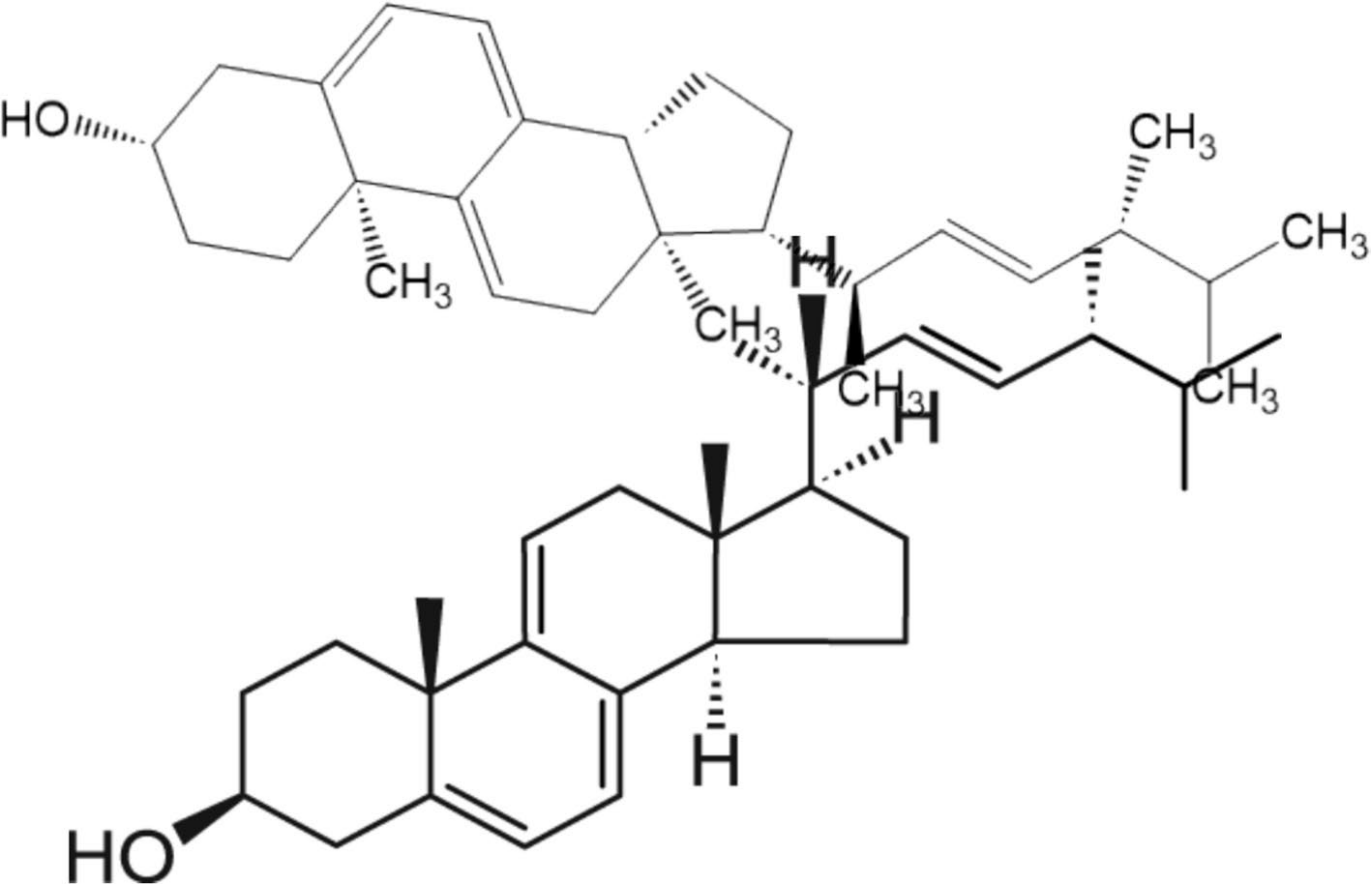
Chemical structure of dehydroergosterol

Dehydroergosterol (DHE) (Fig. 4) is a naturally occurring fluorescent analog with a chemical structure similar to cholesterol and a membrane-embedded behavior and metabolic pathway similar to cholesterol [58]. Intracellularly, DHE can be taken up by cells and incorporated into cell membranes and other organelle membranes like cholesterol. Through its fluorescent properties, the dynamic transport process of cholesterol between organelles can be monitored in a real-time manner [59]. In the study of cholesterol transport mechanisms, DHE has been widely used to follow the transport pathway and time course of cholesterol between different organelles (e.g., endoplasmic reticulum, Golgi apparatus, lysosome) after its uptake from the outside of the cell [60]. It is noteworthy that DHE is similarly transported into recirculating endosomes when present as oleate within low-density lipoprotein (LDL) particles [61]. When cells are supplemented with additional cholesterol, DHE exported from the endocytic network may also undergo esterification at the rough ER membranes and ultimately be stored in lipid droplets (LDs). Quantification of DHE storage in lipid droplets by fluorescence microscopy or quantification of the total esterification level of DHE by high-performance liquid chromatography (HPLC) [62] are both important methods for cholesterol measurement. However, the relatively low fluorescence intensity of DHE, whose fluorescence signal can be difficult to detect at low concentrations or under weak excitation conditions, limits its application in samples with low cholesterol content or high-resolution imaging. In addition, DHE also has the disadvantage of being susceptible to photobleaching, and prolonged exposure to light can lead to attenuation of its fluorescence signal, affecting the accuracy and reproducibility of experimental results [63].

#### BODIPY-cholesterol

To improve the spectral properties of fluorescent sterol analogs, researchers labeled cholesterol with boron dipyrrole methylene (Bodipy) at carbon position 24, resulting in a probe that was 1000-fold brighter than DHE [64]. Boron dipyrrole methylene-labeled cholesterol (BChol) is incorporated into model membranes like cholesterol [65] and behaves similarly in living cells. When added to the plasma membrane, it is readily taken up and translocated into endocytosed vesicles [66]. Due to its high brightness and good photostability, BChol is well suited for studies of diffusion and interaction dynamics using fluorescence correlation microscopy (FCS) [65]. In this regard, Jin et al. [67] evaluated the effect of HCD containing 4% cholesterol on the palatability of fish by adding BODIPY-C16 (0.025%) as a dietary tracer to the diet. The total fluorescence level of the entire digestive tract of the fish was measured after one meal of feeding. It was found that the intestinal lipid fluorescence level of fish fed both diets was similar, indicating that the 4% HCD was as palatable as the standard diet. Blanchard et al. [68] stained postmortem prefrontal cortex (PFC) samples from APOE4 carriers and non-carriers with BODIPY-cholesterol to observe the effects of cholesterol in oligodendrocytes cells, and observed the effects of cholesterol in the PFC and localization of cholesterol in oligodendrocytes. They also found that APOE4 carriers had decreased localization of cholesterol along neurofilaments and increased intracellular accumulation or storage. This study also used BODIPY-cholesterol to stain iPS cell-derived oligodendrocytes to assess cholesterol uptake and intracellular transport. It has been found that BODIPY-cholesterol staining was increased and co-localized with lysosomal co-localization in APOE4 oligodendrocytes, suggesting that cholesterol accumulates in lysosomes.

Although BODIPY cholesterol has many advantages in cholesterol research, it also has some limitations. Its spectral properties, with emission peaks in the ultraviolet band, make detection difficult, requiring special equipment and conditions, and may also pose some risk of photodamage to cells. In addition, the intracellular transport of BChol has been shown to be different from that of natural cholesterol. For example, in the NPC disease model [43], the co-localization of BChol with lysosomal markers is limited under standard assay conditions. Although it can be enriched in lysosomes by prolonged incubation or specific delivery, this delay suggests that it is insufficient to mimic the process of natural cholesterol transport. It may affect the accurate reflection of the dynamics of cholesterol changes in the cell. According to Lehmann et al. [63], the intracellular behavior of BChol analogs may be influenced by their structural properties. For example, analog 6 emits light in the UV and is unstable, making it unsuitable for live cell imaging applications. This also reflects that the structure–function relationship of BODIPY cholesterol markers needs to be further explored to overcome their shortcomings in applications and to improve their accuracy as well as reliability as an analytic tool for cholesterol research.

#### Graphene fluorescent biosensor detection

Graphene fluorescent probes for cholesterol detection is a novel detection method based on the unique optical properties of graphene materials. Graphene is a two-dimensional material composed of a single layer of hexagonal *sp*^2^-hybridized carbon atoms [69]. Due to the hydrophobic properties and regular two-dimensional structure of graphene, it can be combined with the rigid steroid ring structure of cholesterol, resulting in an adsorption interaction between them [70,71]. Through the expressed physical interaction, graphene has been used to reduce the accumulation of cholesterol in lysosomes [72]. Accordingly, Kitko et al. [73] found that graphene would increase the cholesterol content in cell membranes and improve neurotransmission. In addition, Bernabo et al. [70] found that graphene oxide could extract cholesterol from sperm membranes, which had a positive effect on the function of male gametes.

Currently, the commonly used detection method is to perform cholesterol fluorescence detection using sensors based on nitrogen-doped graphene quantum dots/chromium picolinate complexes. For example, Sun et al. [74] combined chromium picolinate (CrPic) and exploited the fluorescence enhancement of N-GQDs/CrPic. They synthesized N-GQDs by a simple hydrothermal method and constructed an eco-friendly cholesterol detection sensor. Since CrPic also acts as a potential receptor for cholesterol through strong affinity and π–π interactions, and the fluorescence of N-GQDs/CrPic is enhanced, it indicates that cholesterol may hinder the transfer of electrons from CrPic to N-GQDs. This N-GQDs/CrPic-based sensor has been successfully applied to the selective determination of cholesterol concentration with a linear range of 0–520 μM and a limit of detection (LOD) of 0.4 μM. However, at present, the technology of graphene fluorescence biosensors is still in the development stage and needs further optimization in terms of specificity.

### Isotope labeling

Isotope labeling techniques are one of the most important tools for studying cellular cholesterol metabolism, including radioisotope labeling (e.g., 3H-cholesterol, 14C-cholesterol) and non-radioisotope labeling (e.g., 15N-cholesterol, 18O-cholesterol). Radioactive cholesterol has been used for decades to study intracellular localization and is still indicated in clinical diagnostics to measure cholesterol efflux capacity in cardiovascular disease prevention [75]. Radioisotope-labeled 3H-cholesterol is chemically and physiologically identical to native cholesterol and can be used to follow processes such as cellular uptake and release of cholesterol [76]. For instance, Tancevski et al. research group [77] labeled J774 macrophages with 3H-cholesterol, injected them into the peritoneal cavity of mice, and measured the activity of the radioactivity in plasma and feces. 3H-cholesterol was found to be increased in the feces of aspirin-treated mice and decreased in plasma, suggesting that aspirin promotes reverse cholesterol transport (RCT) from macrophages to feces. Moreover, Xiao et al. [78] followed the efficiency of cholesterol transport between organelles as well as the direction of transport using 3H-cholesterol. Peroxisomes were purified from cells exposed to 3H-cholesterol and incubated with purified endoplasmic reticulum/microsomes in vitro. It was found that 3H-cholesterol in endoplasmic reticulum increased over time; peroxisomes were found to play a key role in cholesterol transport from lysosomes to ER by 3H-cholesterol translocation assay. Guilbaud et al. [79] used 3H-cholesterol to study the direction of cholesterol transport between organelles in KRASG12D mutant lung tumor-bearing mice and to investigate the role of the cholesterol efflux pathway in tumors. They analyzed the retention and distribution of 3H-cholesterol in lung and peripheral tissues by inhalation and found that there was increased accumulation of 3H-cholesterol in tumor tissue and decreased plasma HDL-C levels. These findings suggested that the cholesterol efflux pathway was impaired in the tumor-bearing mice, which correlated with the development of lung adenocarcinoma. These experiments proposed that 3H-cholesterol radiolabeling enables achieving precise insights into the pathways, and rates of cholesterol transfer between different organelles within a cell, as well as its interactions with other molecules. However, even though these radioisotopes are capable of exhibiting the highest levels of endogenous behavior, their application still poses many hazards in terms of radioactivity. At the same time, the lack of effective methods for real-time detection limits their use to endpoint measurements only [80].

Non-radioisotopically labeled 15N-cholesterol, on the other hand, incorporates mass spectrometry imaging, which provides a new perspective on the study of intracellular cholesterol distribution [81]. During cell culture, 15N-cholesterol is introduced into the cells and after a period of incubation, the cells can be scanned and analyzed at high resolution using mass spectrometry imaging. Mass spectrometry imaging can map the intracellular distribution of 15N-cholesterol based on the signal intensity of ions of different mass numbers, thus visualizing the subcellular localization and distribution of cholesterol within cells [82]. For example, Kraft et al. metabolically labeled live MDCK cells with 18O-cholesterol and 15N-sphingolipids and infected them with H3N2 influenza virus for 24 h. The results were analyzed by high-resolution secondary ion mass spectrometry (HRSIMS). High-resolution secondary ion mass spectrometry (SIMS) and immunolabeling assays were used to assess the co-localization of 18O-cholesterol, 15N-sphingolipids, and influenza virus hemagglutinin proteins and also to investigate whether cholesterol and sphingolipids are enriched at the site of influenza virus assembly and spread. In addition, the same research group are currently working on combining this approach with fluorescence microscopy [83]. Compared to radioisotope labeling, non-radioactive isotope labeling avoids the safety concerns associated with radioactivity and is relatively easy to perform.

### Functionalized cholesterol analogues

Photoactivatable and clickable cholesterol, an innovative tool for cholesterol research, is a novel strategy that combines the advantages of fluorescent cholesterol analogs (easy detection and visualization) with radio-identical analogs (more physiological behavior) by using minimally modified lipid derivatives. It can be easily further functionalized by click chemistry [84–86]. The introduction of acetylenic moieties into lipids allows easy selection and attachment of secondary molecules from a variety of detection, visualization, or affinity markers. Cholesterol derivatives modified in this way are readily taken up and metabolized by living cells. In addition, after immobilization and click-labeling with fluorescent markers, high-resolution imaging is possible [87,88].

Despite these benefits, to effectively utilize affinity labeling in lipid-protein interaction studies, stronger binding between lipids and their interacting proteins is required. This can be achieved by introducing an additional photoactivated diazepine moiety into clickable cholesterol, which is capable of covalently cross-linking even transiently interacting proteins [89]. The utility of such modified cholesterol derivatives for cross-linking was previously demonstrated [90] and used for in vivo identification of interacting partners [91]. Hulce et al. [89] later combined click and photo-cross-linking approaches in a single molecule, allowing them to generate proteomic profiles of cholesterol-protein interactions. By "clicking" biotin onto their cholesterol derivatives, they were able to selectively enrich all cholesterol cross-linked proteins and characterize them by mass spectrometry, providing protein binding site information for a wide variety of cellular and biological process studies.

## Raman technology

### Label-free Raman detection

Raman spectroscopy is a non-destructive method for analyzing the structure and composition of a substance. By observing the spectral features of molecules scattered under light excitation, structural information of a sample can be obtained. Label-free Raman spectroscopy is mainly based on the Raman scattering phenomenon caused by the interaction of laser light with the sample. After the laser beam hit the sample, some of the photons interact with the molecules in the sample, and the scattered photons carry the characteristic frequency of Raman scattering, and the information on the molecules in the sample can be obtained by collecting and analyzing the scattered light. Label-free Raman detection utilizes the Raman spectral signals inherent in the cholesterol molecules themselves and does not require the introduction of additional labeling molecules. Cholesterol molecules produce characteristic Raman scattering signals in specific chemical bond vibrational modes, and detection of these signals enables label-free analysis of intracellular cholesterol. Cheng et al. [92] first used label-free Raman to find CE-specific cholesterol ring vibrations of 702 cm^−1^ in pancreatic cancer tissues and intracellular LDs to indirectly track the LDs. They found a large number of LDs accumulated in the tissues and cells. Subsequently, our group used label-free Raman imaging to differentiate the cellular phenotypes of wild-type (WT) and cell cycle protein-dependent kinase 6 (CDK6) knockout (KO) HeLa cells. By Raman spectroscopy and imaging analysis, it was found that large lipid droplets were formed in KO-type cells. The ratio of saturated/unsaturated fatty acid Raman spectral bands could be used as a Raman spectral marker to distinguish between the two cellular phenotypes, and this study provides the possibility of automatic identification and classification of cellular phenotypes [93]. Lin et al. [94] imaged live Mia PaCa-2 cells with label-free Raman detection of cholesterol to study lipid metabolism. The images were processed by SS-ResNet and the distribution of cholesterol in cell membranes along with lipid droplets was found. The main advantage of label-free Raman detection is that it is non-invasive, does not interfere with the physiological state of cells as well as metabolic processes, and allows real-time monitoring of cholesterol distribution in living cells and tissues under near-physiological conditions. When studying the dynamic changes of cholesterol during cell physiology, label-free Raman assay can provide continuous and realistic information that can help to deeply understand the mechanism of cholesterol in both normal physiological and pathological conditions of cells.

However, the signal intensity of label-free Raman detection is relatively low, which requires high optical resolution and a long acquisition time to obtain sufficient signal intensity for accurate analysis. Researchers have been actively exploring improved methods to carry out experimental studies related to cholesterol using surface-enhanced Raman-based nanoprobes. For example, Jiang et al. [95] used label-free Raman for cholesterol detection by preparing cell membrane-targeted surface-enhanced Raman scattering nanoprobes(SERS nanoprobes), optimizing their Raman signals, and binding them to cholesterol oxidase for the detection of cholesterol in solution, in fixation, and living cells. The results showed that the method could accurately quantify cholesterol with improved signal reproducibility and linearity, which is expected to be used for cholesterol determination in clinical practice. Wu et al. [95] prepared AgNPs@MOF nano enzymes that could catalyze the oxidation of LMG to MG as a SERS substrate. The method detected cholesterol in the linear range of 1.0–100 μM, with a detection limit of 0.36 μM, high specificity. good stability, and reproducibility.

### Label Raman detection

On the other hand, label Raman detection techniques have been developed to overcome the problems of low signal intensity and strong interference in label-free Raman detection. The problem of low signal intensity can be handled by doping cholesterol analogs (e.g., alkyne, bis-alkyne, or deuterium) with strong Raman signal intensity or by using advanced Raman imaging techniques [96]. In addition, unlike fluorescent labeling, Raman labeling is not affected by photobleaching effects and allows continuous imaging of cells [97]. More importantly, the spatial extent of Raman activity patterns is limited to a single or a few chemical bonds, which is much smaller than the fluorescent part of the chemical structure [98].

C–D vibrational probe-based cholesterol and alkyne-based cholesterol analog probes are two common Raman labeling assays. In C–D vibrational probe-based cholesterol, a C–D bond is formed by replacing some of the hydrogen atoms in the cholesterol molecule with heavy hydrogen ones (D). The vibrational frequency of the C–D bond is different from that of the C–H bond, and its characteristic peaks in Raman spectra are located in a specific range of wave numbers, which can improve the signal strength and sensitivity of cholesterol detection. Accordingly, Christian et al. [99] used the D6 cholesterol to observe cholesterol uptake and storage in macrophages. They observed that cholesterol was stored in lipid-like droplet structures at different times of incubation. Alfonso-García et al. [98] used D38-cholesterol labeling to quantify cholesterol in mouse adrenocortical tumor cells. These researchers established a correlation between cholesterol concentration and lipid droplet areas by finding the corresponding Raman peak position of D38-cholesterol (2120 cm^−1^). Although the C–D bond can replace the C–H bond without changing the molecular structure, its Raman intensity is still relatively weak, and an alkyne-based cholesterol analog probe has been developed in which the Raman signal of the alkyne bond is one order of magnitude stronger than that of the C–D bond [100]. The alkyne-based cholesterol analog probe, on the other hand, introduces an alkyne tag on the cholesterol molecule, and the Raman signal of the alkyne group varies from 2000 to 2300 cm^−1^, which does not overlap with other substances in the biological samples. Therefore, the signal of cholesterol—can be detected very clearly [101], which significantly enhances the Raman signal of cholesterol and its quantification. With respect to this approach, Cheng et al. investigated the storage and transport of cholesterol in living cells by the same quantitative method using synthesized phenyl-Diyne cholesterol. They used this linear relationship at the cellular level to project the changes of cholesterol in cells after drug administration for drug screening [97].

Compared with conventional fluorescent probes for intracellular cholesterol detection, alkyne-based Raman labels do not undergo photobleaching, thus allowing prolonged real-time observation. Alkyne-based Raman labels have very strong Raman scattering signals with Raman cross sections several or even tens of times higher than those of conventional fluorescent probes. This finding means that alkyne-based probes have a much higher degree of sensitivity and accuracy in detection. On the other hand, alkyne-based Raman probes have lower cytotoxicity compared to conventional ones with terminal alkyne, which enables using them at higher concentrations without significant cell cytotoxicity [97]. The label Raman detection technique greatly improves the accuracy and reliability of the assay, while maintaining the non-invasive advantages of Raman spectroscopic imaging. These benefits provide a more effective means for high-resolution imaging and quantitative analysis of intracellular cholesterol. The detection of intracellular cholesterol in cancer cells using Raman probes as well as chemometrics for the evaluation of anticancer drugs has demonstrated the validity of this technique in a variety of cancer diseases, such as adrenocortical tumor cells [98] and hepatocellular carcinoma cells [102]. However, the detection of intracellular cholesterol in pancreatic cancer cells using acetylcholesterol probes for drug screening is a gap in current related research.

## Visualization of cholesterol in biological tissues based on mass spectrometry imaging

Mass Spectrometry Imaging (MSI) is a molecular imaging technique based on mass spectrometry analysis technology. It has the advantages of being intuitive, highly sensitive, and highly specific at the molecular level. It can perform label-free imaging of endogenous and exogenous molecules such as small molecular metabolites, proteins, polypeptides, lipids, drugs, and their metabolites in tissue sections or specific tissue microstructures with relatively high spatial resolution. MSI is a powerful tool for studying the spatio-temporal heterogeneity of drug molecules and endogenous biomolecules [103–105]. This approach uses a variety of in situ ionization techniques to ionize compounds on the sample surface, transfer them to the mass spectrometer to obtain the mass-to-charge ratio (*m*/*z*) and ion intensity of the ions. Then, it uses mass spectrometry imaging software to convert the mass spectrometry data sets containing spatial position information into ion distribution images, thereby visualizing the spatial distribution characteristics of the substances being measured. According to the differences in ionization techniques, the three commonly used MSI modalities at present are matrix-assisted laser desorption/ionization mass spectrometry imaging (MALDI–MSI), desorption electrospray ionization mass spectrometry imaging (DESI–MSI), and secondary ion mass spectrometry imaging (SIMS–MSI) [106].

### Matrix-assisted laser desorption/ionization mass spectrometry imaging (MALDI–MSI)

MALDI–MSI was officially introduced by the Caprioli research team in 1997 and soon became an effective and reliable technique [107]. The MALDI–MSI technique uses matrices capable of absorbing lasers of specific wavelengths to form co-crystals with the molecules on the sample surface. Laser irradiation causes in situ desorption and ionization of the compounds to be measured. It is characterized by a wide mass range, high sensitivity, high spatial resolution (5–50 µm), and a strong ability to resist against interference from impurities [108]. The choice of the MALDI matrix is one of the most important aspects determining the quality of the data [109]. Most of the traditional MALDI matrices are small organic molecules, such as 2,5-dihydroxybenzoic acid (DHB), α-cyano-4-hydroxycinnamic acid (CHCA), 9-aminoacridine (9-AA) and sinapic acid (SA) [106]. There have been reports on the use of MALDI-MSI to study and analyze the detection of various biomolecules (e.g., lipids, proteins) and drug molecules in both positive and negative ion modes [106,110,111]. The research group led by Shi [112] established a MALDI-MSI method for direct and rapid analysis of the spatial distribution of cholesterol in Alzheimer’s disease (AD) mouse models, cancer tissues, and organs. This method determined the cholesterol content in the cerebellar AD model mice and the wild-type (WT) group, demonstrating that cholesterol can serve as a potential diagnostic marker for AD. Meanwhile, the analysis of the hydrocarbon fragments of cholesterol by MS/MS provided valuable data and strong evidence for the law of cholesterol mass spectrometry fragmentation and the accurate analysis of complex systems by mass spectrometry, successfully achieving direct imaging analysis of cholesterol by MALDI–MSI. However, in the low relative molecular mass range (*m*/*z* < 500 Da), traditional small molecule matrices tend to generate matrix peaks and have a relatively high ion suppression effect, which interferes with the mass spectrometric response of the compounds being measured. Researchers are currently working to develop new matrices and improve instrument performance.

### Desorption electrospray ionization mass spectrometry imaging (DESI–MSI)

Desorption electrospray ionization mass spectrometry imaging (DESI–MSI) is an electrospray-based ion source developed by Takats et al. in 2004 [113]. In the DESI technique, a beam of charged droplets, formed by pneumatically assisted electrospray, is sprayed onto the sample surface. The solvent in the droplets immediately undergoes extraction and dissolution processes with the substances to be measured on the sample surface. As the droplets bounce off the surface, they form finer secondary droplets. With the rapid evaporation of the solvent, the charge is transferred to the molecules of the substances to be measured, providing the desorption and ionization of the molecules on the sample surface [106]. DESI–MSI has been widely used in cancer margin analysis, biomarker discovery in diseased tissues, drug discovery and development, and neuroscience research [114]. Compared to MALDI–MSI, DESI–MSI is an open mass spectrometry imaging technique. It is easy to use and the sample processing procedure is simple. It is often used to detect small molecules in the low relative molecular mass range (*m*/*z* < 1000 Da) [106]. DESI imaging has been used to localize cholesterol sulfate in prostate cancer and normal tissue sections [115]. Cholesterol sulfate has been identified as a compound that can distinguish cancer tissue from normal one. This compound is almost exclusively found in cancerous tissue, making it a promising biomarker candidate and a tool for diagnosing prostate cancer. Pan et al. [116] performed comprehensive imaging of polar/non-polar lipids by DESI as well as DESI–PI and analyzed data from multiple samples. They found that cholesterol is the most important biomarker in melanocytic nevi, indicating that DESI–PI MSI has great value in the intraoperative diagnosis of skin and other diseases (such as melanoma). In addition, laser ablation inductively coupled plasma mass spectrometry imaging (LA–ICP–MSI) can quantitatively characterize the tissue distribution of different inorganic elements or different isotopes of the same element. This technique is suitable for studying the aggregation characteristics of metal elements in the disease pathogenesis and for revealing the distribution, transport, and clearance processes of metal nanomedicines in living organisms [117].

### Secondary ion mass spectrometry imaging (SIMS–MSI)

The secondary ion mass spectrometry imaging (SIMS–MSI) technique uses a focused primary ion beam to bombard the sample surface, with sputtering to produce positive and negative secondary ions. As the energy of the primary ion beam is relatively high and on the other hand, biological macromolecules such as lipids, polypeptides, and proteins are prone to fragmentation, it is usually used for elemental analysis [118]. In recent years, with the development of new cluster ion beams (such as C60+), sample surface damage and molecular fragmentation have been significantly reduced, and SIMS-MSI is gradually being applied for studies of tissue distribution of metabolites and information exchange between colonies [119]. The SIMS-MSI technique has an extremely high spatial resolution, which can reach the subcellular level (about 50 nm), and has unique advantages in longitudinal depth analysis [120,121]. Currently, high-resolution microscopy imaging technology can be combined with isotope tracer technology known as nanoscale secondary ion mass spectrometry (Nano-SIMS). Nano-SIMS offers unprecedented opportunities to study the composition and metabolic characteristics of microbial communities in complex environmental samples at the single-cell imaging level. Its extremely high sensitivity and accuracy give it an advantage over other single-cell research methods [122]. For instance, Jiang et al. [122] used Nano-SIMS to observe the movement of cholesterol in and out of cells and visualized and quantified the cell surface cholesterol pool, the so-called “accessible cholesterol”. Using bacterial protein-binding Nano-SIMS imaging, the researchers showed that these accessible cholesterol pools are not evenly distributed across the plasma membrane of the cell, but are highly enriched in the specific protrusions of the plasma membrane microvilli. In addition, Tontonoz’s research group [123] used nanoscale secondary ion mass spectrometry (Nano-SIMS) imaging technology to visualize the uptake and distribution of cholesterol by intestinal epithelial cells. Therefore, nano-SIMS imaging provides a unique perspective on the distribution of cholesterol across the plasma membrane. Future studies will enable to evaluate the mechanisms by which cells deal with excess cholesterol, better understand the mechanisms of cholesterol movement in cells as well as tissues, and develop new strategies to reduce blood cholesterol levels.

MSI has become one of the most important analytical tools in drug discovery as a label-free, highly specific, and sensitive visualization technique. The new spatial chemical information provided by MSI will play an important role in pharmacokinetics-pharmacodynamics, cellular pharmacokinetics, and drug delivery, as well as in vivo and in vitro drug evaluation studies in different contexts like organoids and 3D cell models. Over the past decade, the MSI technique has been associated with significant improvements in detection sensitivity, quantitative capability, analytical throughput, and data analysis speed. With further development and improvement, the MSI technique will definitely demonstrate more capabilities and broader application prospects in drug discovery in the future [126].

Different aspects of cholesterol detection methods including their principle, advantages, limitations, and applications are summarized and listed in Table 1.

**Table 1:** Comparison of cholesterol detection methods

Detection method	Principle	Advantages	Limitations	Applications	References
SREBP2 Activity-Based Assay	Measures cholesterol levels via SREBP2 activation through proteolysis and nuclear translocation	High sensitivity; suitable for monitoring intracellular cholesterol levels dynamically	Relies on ER cholesterol fluctuations; lacks spatial resolution	Applicable for studies on cholesterol metabolism and biosynthesis	[10–18]
Gas Chromatography/Liquid Chromatography (GC/LC–MS)	Separates and quantifies cholesterol and esters using chromatography coupled with mass spectrometry	High sensitivity and specificity; ideal for small sample volumes	Complex sample preparation; lacks spatial information and is time-consuming	Suitable for endpoint quantification of cholesterol in serum or cell extracts	[17,19,20,22,23]
Enzymatic Assay	Uses cholesterol oxidase to produce hydrogen peroxide, detected via fluorescence or colorimetry	Simple operation; high sensitivity; widely used for cholesterol quantification in serum	Susceptible to interference, reducing accuracy in complex samples	Widely used for clinical and routine cholesterol quantification in serum	[25–31]
Modified Abell–Kendall Method	Uses saponification, solvent extraction, and colorimetric reaction for cholesterol quantification	High accuracy; serves as a reference method	Involves corrosive reagents and complex protocols, making it unsuitable for high-throughput	Primarily used for method calibration or laboratory studies	[32–34]
Filipin Staining	Binds free cholesterol with Filipin to produce fluorescence, visualizing intracellular cholesterol	High spatial resolution; suitable for fixed sample analysis	Low fluorescence intensity, photobleaching; toxic to live cells	Useful for analyzing free cholesterol distribution in cell membranes or lysosomes	^[[35–40]]^
PFO-D4 Probe	Targets cholesterol-enriched membrane regions using fluorescently labeled PFO derivatives	High sensitivity; compatible with super-resolution microscopy	Potentially disturbs cellular structures; may miss certain cholesterol regions	Suitable for studying dynamic cholesterol distribution and high-resolution imaging	[44,78–83,92,93]
DHE Fluorescent Analogue	Monitors dynamic cholesterol transport and distribution via the natural fluorescence of dehydroergosterol (DHE)	Biocompatible; enables real-time tracking of cholesterol dynamics	Low fluorescence intensity; prone to photobleaching	Ideal for real-time monitoring of cholesterol transport and distribution	[58–62]
BODIPY-Cholesterol	Labels cholesterol with BODIPY fluorophores for imaging and dynamic monitoring	Bright fluorescence; good photostability; suitable for dynamic studies	Transport behavior differs from natural cholesterol	Suitable for intracellular cholesterol monitoring and transport studies	[43,63–68]
Graphene Fluorescent Probes	Detects cholesterol via fluorescence quenching/recovery properties of graphene combined with aptamers	Rapid detection; suitable for high-throughput analysis	Specificity requires optimization; the biocompatibility of graphene needs further validation	Suitable for rapid cholesterol quantification; has potential but requires further refinement	[95,124]
Functionalized Cholesterol Analogues	Labels cholesterol using click chemistry or photoactivation for fluorescence and mass spectrometry analysis	Combines fluorescence visualization with molecular labeling; supports high-resolution studies	Photoactivated derivatives may interfere with cellular processes	Useful for studying cholesterol-protein interactions and tracking dynamic changes at high resolution	[84–91]
Label-Free Raman Spectroscopy	Leverages intrinsic Raman signals of cholesterol molecules for label-free quantification and distribution	Non-invasive; suitable for real-time monitoring in live cells and tissues	Weak signal intensity; requires optimization of resolution and acquisition time	Suitable for dynamic distribution and metabolic studies, particularly in near-physiological conditions	[92–94]
Raman Tagging	Enhances Raman signals using alkynyl or deuterium tags for quantitative and dynamic distribution analysis	Strong Raman signals; high sensitivity; resistant to photobleaching	Tags may alter natural cholesterol behavior	Ideal for high-resolution imaging and quantitative analysis of cholesterol, especially for metabolic studies	[65,96–101]
MALDI-MS Imaging	Uses matrix-assisted ionization to analyze cholesterol spatial distribution in tissue sections	High spatial resolution imaging; capable of detecting multiple molecules simultaneously	Requires fixed samples; cannot monitor dynamic changes	Suitable for analyzing cholesterol distribution in tissue sections or fixed samples	[107,110–112,125–128]
DESI-MS Imaging	Employs desorption electrospray ionization for mapping cholesterol and its metabolites	Requires no matrix; ideal for low-molecular-weight compounds	Limited spatial resolution; suitable for fixed samples only	Useful for analyzing metabolic biomarkers in pathological samples, e.g., prostate cancer	[113,115,116,129]
Nano SIMS	Uses high-energy ion beams to stimulate cholesterol isotope-labeled molecules, providing high-resolution imaging	High sensitivity; suitable for single-cell level analysis	Requires labeled samples; complex detection process	Ideal for studying cholesterol distribution and dynamics at single-cell or subcellular levels	[122,130]

## Conclusion and future perspectives

This review presents a comprehensive overview of the primary techniques used for determining cellular cholesterol content, including chromatography-based methods, fluorescent probes, Raman spectroscopy, and mass spectrometry imaging (MSI). Each of these methodologies brings unique strengths—chromatography offers high sensitivity and reliability; fluorescent probes allow for dynamic monitoring; Raman techniques provide chemical specificity; and MSI delivers unmatched spatial resolution. Traditional methods such as gas chromatography and enzymatic assays remain widely used due to their robustness and accuracy, while newer approaches like MSI and Raman-labeled detection offer significant potential in imaging and real-time tracking applications.

As the field advances, cholesterol measurement is expected to evolve in several key directions. First, integrative approaches will combine the complementary strengths of existing methods—for example, the dynamic monitoring ability of fluorescent probes with the spatial precision of MSI—to develop hybrid tools with broader applicability. Second, high-throughput and automated systems, driven by microfluidic platforms and artificial intelligence, are anticipated to meet the growing demand for large-scale screening and clinical diagnostics. Third, nanotechnology is likely to enhance sensitivity and specificity through the development of nanoscale sensors and markers. Finally, in vivo dynamic monitoring remains a critical frontier, where label-free or minimally invasive techniques may allow real-time observation of cholesterol behavior in living systems. These innovations will not only expand analytical capabilities but also deepen our understanding of cholesterol metabolism in health and disease.

Specificity—the ability to selectively detect cholesterol without interference from structurally similar molecules—is fundamental to reliable measurement. Among the reviewed methods, chromatographic techniques (GC and LC) offer high specificity by physically separating cholesterol from other sterols before quantification. Enzymatic assays, though user-friendly and widely adopted, may suffer from interference by endogenous compounds or cross-reactivity with non-cholesterol sterols. Fluorescent probes, such as filipin III, bind selectively to free cholesterol but may also interact with other lipids, limiting their precision in certain tissue contexts. Cholesterol-dependent cytolysins, like PFO-based probes, enhance membrane specificity but can compromise live-cell integrity.

Several fluorescent analogs have been developed to study cholesterol transport and localization. Dehydroergosterol (DHE) closely mimics cholesterol’s biophysical properties but suffers from weak fluorescence and photobleaching. BODIPY-cholesterol improves brightness and stability but may not replicate natural intracellular trafficking under disease conditions. Graphene-based biosensors, though still in early development, utilize physical interactions for promising specificity but require further validation. Isotope-labeled cholesterol—either radioactive or stable—offers unmatched accuracy by preserving the molecule’s native behavior, though practical limitations such as safety, cost, and imaging demands exist. Clickable or photoactivatable analogs enable detailed protein-interaction studies, but chemical modifications may subtly alter biological behavior, requiring rigorous controls.

Raman-based techniques provide additional options for high-specificity detection. Label-free Raman exploits the molecule’s inherent vibrational signatures (e.g., 702 cm⁻^1^ ring vibrations), while labeled approaches introduce unique vibrational tags—such as alkynes—that resonate in cell-silent regions (2000–2300 cm⁻^1^), enhancing signal clarity and eliminating background noise. Mass spectrometry imaging (MSI)—including MALDI-MSI, DESI-MSI, and SIMS/Nano-SIMS—stands out by directly measuring cholesterol’s mass-to-charge ratio and fragmentation profile, enabling highly specific and spatially resolved detection even in complex tissues.

Despite these advances, no single technique achieves perfect specificity across all scenarios. Therefore, understanding the inherent strengths and limitations of each method—and strategically combining complementary approaches when appropriate—is essential for achieving accurate, reproducible, and meaningful cholesterol measurements in both experimental and clinical settings.

## Data Availability

Not applicable.
